# Bronchovascular injury associated with clinically significant hemoptysis after CT-guided core biopsy of the lung: Radiologic and histopathologic analysis

**DOI:** 10.1371/journal.pone.0204064

**Published:** 2018-09-21

**Authors:** Heekyung Kim, Dohee Kwon, Soon Ho Yoon, Hyungjin Kim, Chang Min Park, Jin Mo Goo, Yoon Kyung Jeon, Su Yeon Ahn

**Affiliations:** 1 Department of Radiology, Seoul National University College of Medicine, Seoul, Korea; 2 Department of Pathology, Seoul National University College of Medicine, Seoul, Korea; 3 Institute of Radiation Medicine, Seoul National University Medical Research Center, Seoul, Korea; University of Western Ontario, CANADA

## Abstract

**Objective:**

To evaluate bronchovascular injuries as the causative occurrence for clinically significant hemoptysis after percutaneous transthoracic needle biopsy (PTNB).

**Materials and methods:**

We included 111 consecutive patients who experienced hemoptysis after cone beam CT (CBCT)-guided PTNB from January 2014 through January 2017. Clinically significant hemoptysis was defined as hemoptysis causing hemodynamic instability or oxygen desaturation greater than 10% of baseline. The lesion characteristics were evaluated on CT images. The penetration of bronchovascular structures along the trajectory of the introducer needle and potential penetration at the firing of the biopsy gun were assessed on CBCT images. The cutting injury of bronchovascular structures was histopathologically assessed in biopsy specimens. The associated factors for clinically significant hemoptysis were assessed using logistic regression analyses.

**Results:**

Seventeen patients (15.3%; 95%CI, 9.7%-23.2%) had clinically significant hemoptysis. On univariate analysis, the open bronchus sign (*P* = .004), nodule consistency (*P* = .012), potential penetration of a pulmonary vessel or bronchus 1 mm or larger at firing (*P* = .008 and *P* = .038, respectively), and a cutting injury of a pulmonary vessel 1 mm or larger (*P* = .007) or a bronchial structure (*P* = .041) were associated with clinically significant hemoptysis. Multivariate analysis found the following significant associated factors: potential penetration of a pulmonary vessel 1 mm or larger at firing (OR, 3.874; 95%CI, 1.072–13.997; *P* = .039) and cutting injury of a pulmonary vessel 1 mm or larger (OR, 6.920; 95%CI, 1.728–27.711; *P* = .006) or a bronchial structure (OR 4.604; 95%CI, 1.194–17.755; *P* = .027).

**Conclusion:**

Potential penetration and cutting injury of bronchovascular structures 1mm or larger at firing were independently associated with clinically significant hemoptysis after PTNB.

## Introduction

Percutaneous transthoracic needle biopsy (PTNB) has been considered a crucial diagnostic procedure for the pathologic evaluation of peripheral pulmonary lesions in that it is less invasive than surgical lung biopsy and similar to higher diagnostic accuracy than bronchoscopic biopsy [1]. With the introduction of advanced computed tomography (CT)-based imaging modalities for needle guidance, the diagnostic accuracy of PTNB has been reported to be as high as 97% [2]. The role of PTNB has been increasing recently for the mutational analysis of non-small cell lung cancer in the era of targeted therapy [3].

Although PTNB is a safe procedure and is less invasive than surgical lung biopsy, it is inevitably accompanied by a risk of procedure-related complications. A recent meta-analysis reported that the minor and major complication rates were 38.3% (95%CI, 34%-343.5%) and 5.7% (95%CI, 4.4%-7.4%) after CT-guided core biopsy, respectively [4]. Among the PTNB-related complications, hemoptysis occurred in 4.1% (95%CI, 2.8%-6.1%) of patients after PTNB, with particularly higher risks among the patients with small deep-seated lesions, or lesions with a subsolid composition, patients undergoing dual-antiplatelet therapy, and those with emphysema, older age, and female gender [4–9]. Even though post-PTNB hemoptysis is usually self-limited [10], hemoptysis can be massive and potentially fatal [11]. The occurrence of an injury to a bronchus or pulmonary vessel during the PTNB procedure has been suggested to be a potential source of fatal hemoptysis [12], but has not been systematically analyzed associated with clinically significant hemoptysis.

Thus, the purpose of this study was to evaluate bronchovascular injuries as the causative occurrence for clinically significant hemoptysis after PTNB.

## Materials and methods

This retrospective study was conducted with the approval of our institutional review board of Seoul National University Hospital (IRB No. H1704-022-843). The requirement for informed consent from patients was waived given the anonymous data analysis of this study.

### Study population

**Fig 1:**
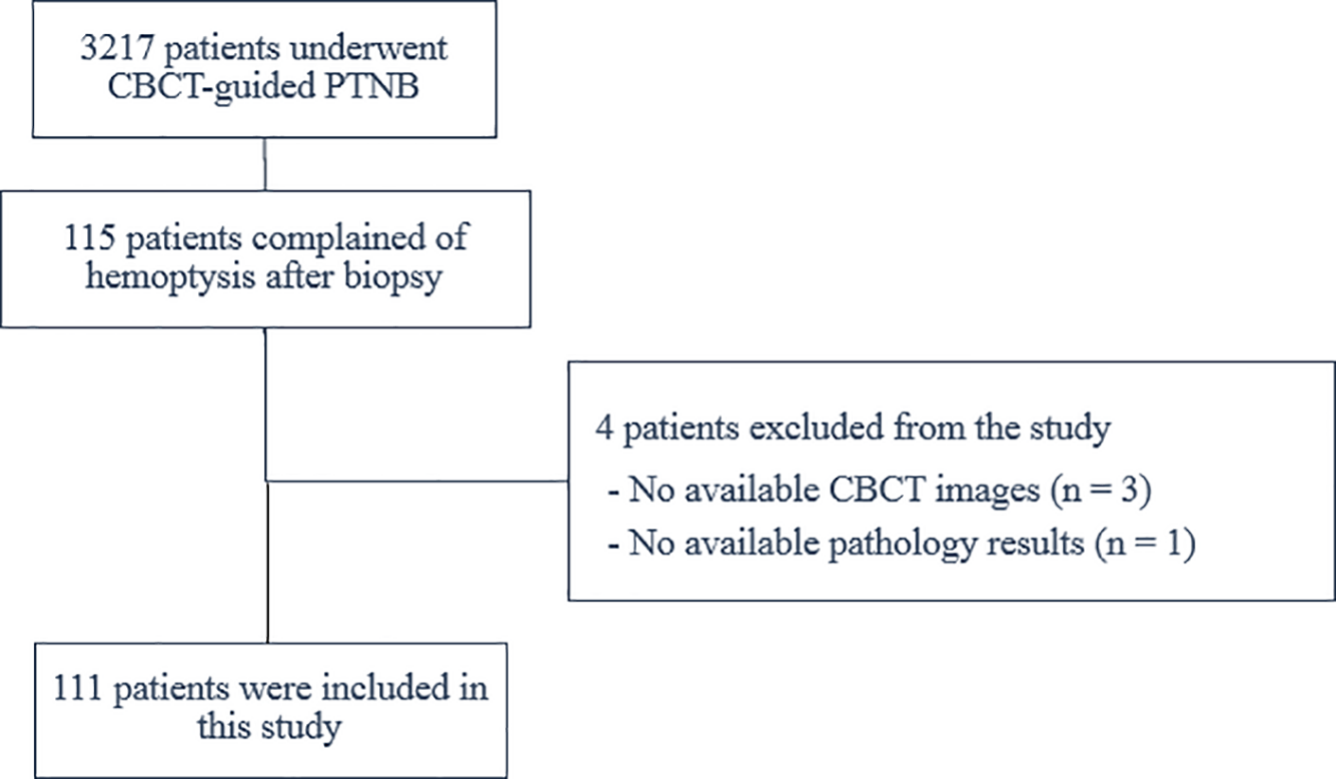
Study diagram for patient inclusion.

Among 3217 patients who underwent cone-beam CT (CBCT)-guided PTNB from January 2014 through January 2017, 115 consecutive patients complained of hemoptysis after the PTNB (hemoptysis incidence, 3.57%). We excluded 3 patients who did not have available CBCT images on a picture archiving and communication system and 1 patient who did not have any available pathology results. Finally, a total of 111 patients (mean age 64.7 years; age range, 41–74 years; 57 male and 54 female) were included in this study (Fig 1).

One author (H.K.K.) reviewed the baseline characteristics of the patients, including their smoking history, past medical history, and coagulation profiles. According to our departmental policy, PTNB was not performed in patients with the following abnormal coagulation profiles: a prothrombin time greater than an international normalized ratio of 1.5, an activated partial thromboplastin time longer than 50 seconds, or a serum platelet count of less than 50 × 10^3^/μL. We recommended discontinuing anti-platelet agents or anticoagulants for a week before PTNB if the referred patient was taking such medications.

### CBCT-guided PTNB procedure

During the study period, we used 2 different CBCT systems: 88 patients, Allura Xper FD20 (Philips Healthcare, Best, the Netherlands); 23 patients, AXIOM Artis dTA (Siemens, Erlangen, Germany). CBCT scans were performed at least 3 times in either the supine or prone position during the PTNB procedure: first, pre-procedural CBCT scanning before needle insertion to localize the pulmonary lesion and to plan the needle path; second, intra-procedural CBCT scanning after needle insertion to confirm the placement of the introducer needle before firing the biopsy gun; and third, post-procedural CBCT scanning after removing the needle to check for post-procedural complications. The acquired CBCT images were transferred to a dedicated workstation (X-Leonardo, Siemens Healthcare; XtraVision, Philips Healthcare). The CBCT images were reconstructed in axial, coronal, and sagittal planes with a 1mm slice thickness.

PTNB was performed either by 1 of 2 dedicated thoracic radiologists (C.M.P and S.H.Y) or by fellows under the supervision of the radiologists. All cases underwent a core biopsy. A 20-gauge semiautomated cutting needle with a 19-gauge coaxial introducer (Stericut, TSK Laboratory) was used in 87 patients, and an 18-gauge semiautomated cutting needle with a 17-gauge coaxial introducer (Stericut, TSK Laboratory) was used in 24 patients. The active cutting needle part of the needles was 22 mm in length, regardless of the gauge. On the pre-procedural CBCT images, the thoracic radiologist drew the safest and effective trajectory needle path to the lesion, trying to avoid bullae, or fissure. Patients were instructed to hold their breath when the needle passed through the pleura, or moved in the lung parenchyma. After confirming the location of the needle tip on intra-procedural CBCT images, the radiologist removed the stylet of the introducer needle while the patient performed a breath-hold maneuver and inserted the cutting needle into the outer sheath of the introducer. After performing the biopsy, once absence of hemoptysis was confirmed, the patient`s position was rapidly changed to place the biopsy site on the dependent side. The patient was closely observed for 3 hours. The patient who complained of hemoptysis was instructed to assume a lateral position and encouraged to expectorate the aspirated blood. If oxygen saturation decreased on pulse oximetry, oxygen was delivered via a nasal cannula.

Hemoptysis was assessed by the practitioner’s records of the procedure and the nursing records that were made during the procedure. Massive hemoptysis is classically defined as 600 mL or more of hemoptysis within 24 hours [13], but the amount of hemoptysis was difficult to measure precisely during the urgent management and clinically significant hemoptysis accompanies abnormal gas exchange or hemodynamic instability [14]. Accordingly, we defined clinically significant hemoptysis as hemoptysis causing hemodynamic instability as defined when a systolic blood pressure <90mmHg or decreased in systolic blood pressure >40mmHg [15], or an oxygen desaturation greater than 10% from the baseline level of oxygen saturation on continuous arterial oxygen saturation monitoring by pulse oximetry.

We collected the following baseline characteristics of PTNB procedures: the number of biopsy specimens acquired, the distance between the pleura to the lesion in the position assumed by the patient, emphysema, needle gauge, needle insertion time (min), and the relative position of the introducer needle tip on the lesion on intra-procedural CBCT images. The tip position was categorized into 3 groups, depending on whether the tip was located in the center of the lesion, abutted onto the margin of the lesion, or the tip was located outside the lesion.

### CT analysis of lesion characteristics

The characteristics of the pulmonary lesions that were referred for PTNB were reviewed on pre-procedural chest CT images by 2 thoracic radiologists (H.K.K. and S.H.Y., with 5 and 12 years of clinical experience of chest CT interpretation, respectively) through consensus blinded to the results of hemoptysis. The mean interval between pre-procedural chest CT and PTNB was 21.3±17.7 days. The lesion characteristics included the longest axial diameter, lesion composition, and presence of cavitation and necrosis. The lesions were categorized by composition as sub-solid nodules and solid nodules. Sub-solid nodules were defined as nodules containing any ground glass opacity within the lesion [16]. In addition, we analyzed the presence of the open bronchus sign, which was defined as an intra-lesional bronchiolar dilatation that continued to a proximal bronchus with a preserved luminal patency [17].

### Analysis of bronchovascular injuries during the procedure

Bronchovascular injuries during the PTNB procedure were defined as encompassing penetrating injuries by the needle and cutting injuries by the biopsy gun. Penetrating injuries can occur along the path of needle insertion and at the firing of biopsy gun by the advancement of the cutting needle. Two radiologists (H.K.K. and H.K.) independently evaluated these two kinds of penetrating injuries in a qualitative manner on pre- and intra-procedural CBCT images blinded to the results of hemoptysis. Any disagreement was resolved by consensus. The penetrating injury along the path of needle insertion was confirmed by the presence of a bronchovascular structure 1 mm or larger in diameter bi-dimensionally placed along the path of the inserted introducer needle on intra-procedural CBCT images. If needed, the tracing of bronchovascular structures was supported by a simultaneous review of pre-procedural and intra-procedural CT images on a side-by-side display. To evaluate penetrating injury by the advancement of the cutting needle after firing, the readers evaluated whether a bronchovascular structure 1 mm or larger in diameter was bi-dimensionally placed along the expected needle path of the biopsy gun within 22 mm of length at firing. If the bronchovascular structure was bi-dimensionally placed within the expected path, it was labeled as potential penetration rather than as penetration, because we could not confirm the post-biopsy penetration on intra-procedural CT images that were taken prior to firing although those bronchovascular structures were at risk for penetration.

Cutting injuries of bronchovascular structures were assessed based on a histopathologic evaluation of the presence of bronchi or bronchial vessels in the biopsy specimen by a single pathologist (D.K.) blinded to any clinical information. The maximum diameter of the vessels in the biopsy specimen was measured and classified into 5 grades: grade 0 was defined as no vessels included in the biopsy specimen; grade 1, vessels with a maximum diameter less than 0.5 mm; grade 2, between 0.5 mm and 1 mm; grade 3, between 1 mm and 1.5 mm; grade 4, between 1.5 mm and 2 mm; and grade 5, vessels with a diameter greater than 2 mm. Findings of grade 3 or higher were considered to indicate a cutting injury of a pulmonary vessel. Depending on whether the bronchial structures were included in the biopsy specimens, the groups were divided into 3 grades: grade 0, the absence of bronchial structures; grade 1, containing only a bronchial structure without cartilage; grade 2, containing a bronchial structure with cartilage) (S1 and S2 Figs). Samples containing a bronchial structure with or without cartilage were considered to indicate a cutting injury of the bronchial structure.

### Statistical analysis

Univariate logistic regression analysis was used to analyze the factors affecting the incidence of clinically significant hemoptysis. Multivariate logistic regression analysis was then performed of factors found to have a *P*-value < .05 in the univariate analysis to identify independent association with clinically significant hemoptysis. Interobserver agreement for the evaluation of the 2 kinds of penetrating injuries in a qualitative manner on pre- and intra-procedural CBCT images was determined by Cohen kappa statistics, with agreement defined as very good (kappa, >0.8), good (kappa, 0.61–0.8), moderate (kappa, 0.41–0.6), fair (kappa, 0.21–0.4), or poor (kappa, ≤0.2) [18]. *P*-values < .05 were considered to indicate statistical significance. Statistical analysis was performed using SPSS version 23 (IBM Corp., Armonk, NY, USA).

## Results

Among the 111 patients with hemoptysis who were included in this study, 17 (15.3%; 95%CI, 9.7%-23.2%) had clinically significant hemoptysis, causing oxygen desaturation greater than 10% from baseline (n = 15) or hemodynamic instability (n = 4). The incidence of clinically significant hemoptysis was 0.53% (17 of 3217; 95%CI, 0.33%-0.85%) in patients who underwent PTNB. Incidence of pulmonary hemorrhage more than lobar or hemothorax detected on post-procedural CT occurred in 5 patients.

In regards to bronchovascular injuries, penetration of either pulmonary vessels or a bronchus 1 mm or larger was found in 61.3% of patients (pulmonary vessels, 60.4%; bronchus, 16.2%), during the placement of the introducer needle on pre-procedural CT images. On the intra-procedural CT images, the potential penetration of either pulmonary vessels or a bronchus 1 mm or larger at firing was identified in 46.8% of patients (pulmonary vessels, 39.6%; bronchus, 17.1%). Inter-observer agreement was good for assessing penetrating injuries or potential injuries of bronchovascular structures (kappa coefficient range, 0.681–0.748) on the pre- and intra-procedural CBCT images.

Of the 111 patients included in the study, 5 biopsy specimens were not available and cannot be reviewed. On the histopathologic examination, cutting injuries of either pulmonary vessels 1 mm or larger or bronchial structures were found in 47.2% of patients (pulmonary vessels, 25.5%; bronchus, 26.4%). The mean diameter of the cut bronchial structures was 0.7 mm. In patients with clinically significant hemoptysis, the diameters of the cut pulmonary vessels and bronchial structures were between 1.0 and 1.5 mm and 1.5 mm, respectively. All 17 patients with clinically significant hemoptysis had at least one of penetrating injury (n = 10), potential penetrating injury (n = 13), or cutting injury of bronchovascular structures (n = 14).

In the univariate analysis, a subsolid nodule (*P* = .012), the open bronchus sign (*P* = .004), and potential penetration of a pulmonary vessel or bronchus 1 mm or larger at firing (*P* = .008 and *P* = .038, respectively) were significant factors associated with clinically significant hemoptysis. In addition, the presence of a cutting injury of pulmonary vessels 1 mm or larger or bronchial structures in the biopsy specimen was more frequently detected in patients with clinically significant hemoptysis (*P* = .007 and *P* = .041, respectively)(Table 1).

**Table 1:** Results of univariate analysis to determine influencing factors for severity of hemoptysis.

	Clinically significant hemoptysis (n = 17)	No clinically significant hemoptysis (n = 94)	OR	95% CI	p value
**Clinico-laboratory factors**					
** Male (vs female)**	64.7% (11/17)	48.9% (46/94)	1.913	0.654–5.598	0.236
** Age ≥ 60 (vs < 60)**	52.9% (9/17)	64.9% (61/94)	0.609	0.215–1.726	0.350
** Smoking (vs non-smoker)**	58.8% (10/17)	38.3% (36/94)	2.302	0.804–6.588	0.120
** Patient Medical history (vs none)**					
** History of pulmonary TB**	52.9% (9/17)	45.7% (43/94)	2.930	0.589–14.577	0.189
** History of liver cirrhosis**	5.9% (1/17)	2.1% (2/94)	7.000	0.427–114.701	0.173
** History of cancer**	29.4% (5/17)	22.3% (21/94)	3.333	0.588–18.891	0.174
** Anticoagulation medication (vs none)**	5.8% (1/17)	2.1% (2/94)	2.875	0.246–33.600	0.400
** Prothrombin time (international normalized ratio) ≥ 1.0**	41.2% (7/17)	43.6% (41/94)	0.905	0.317–2.582	0.852
** Activated partial thromboplastic time(s) ≥ 35**	17.6% (3/17)	23.4% (22/94)	0.701	0.185–2.666	0.602
** Platelet count (x10**^**3**^**/μL) < 100**	5.9% (1/17)	1.1% (1/94)	5.812	0.346–97.722	0.222
**CT lesion characteristics**					
** Target size (mm) < 20**	52.9% (9/17)	33.0% (31/94)	2.286	0.804–6.500	0.121
** Emphysema (vs none)**	76.5% (13/17)	84.0% (79/94)	1.621	0.465–5.652	0.449
** Subsolid nodule (vs solid)**	35.3% (6/17)	10.6% (10/94)	4.582	1.392–15.081	0.012
** Cavity or necrosis (vs none)**	11.8% (2/17)	5.3% (5/94)	2.373	0.421–13.369	0.327
** Open bronchus sign (vs none)**	58.8% (10/17)	22.3% (21/94)	4.966	1.685–14.637	0.004
**Biopsy procedural factors**					
** Distance between the pleura to the lesion (mm) ≥ 40**	23.5% (4/17)	26.6% (25/94)	0.849	0.253–2.849	0.791
** Prone position (vs supine)**	70.6% (12/17)	61.7% (58/94)	1.490	0.485–4.580	0.487
** 18G needle gauze (vs 21G)**	35.3% (6/17)	19.1% (18/94)	2.303	0.752–7.055	0.144
** Needle insertion time (min) ≥ 10**	11.8% (2/17)	21.3% (20/94)	0.493	0.104–2.336	0.373
** Number of acquisition of biopsy specimen ≥ 3**	17.6% (3/17)	40.4% (38/94)	0.316	0.085–1.174	0.085
** Introducer needle tip outside the lesion**	35.3% (6/17)	23.4% (22/94)	2.433	0.826–7.172	0.107
**Bronchovascular injuries**					
** Penetration of vessel 1mm or larger along the inserted needle path (vs none)**	58.8% (10/17)	60.6% (57/94)	0.927	0.324–2.652	0.888
** Penetration of bronchus 1mm or larger along the inserted needle path (vs none)**	29.4% (5/17)	13.8% (13/94)	2.596	0.785–8.588	0.118
** Potential penetration of vessel 1mm or larger when fired (vs none)**	70.6% (12/17)	34.0% (32/94)	4.650	1.506–14.354	0.008
** Potential penetration of bronchus 1mm or larger when fired (vs none)**	35.3% (6/17)	13.8% (13/94)	3.399	1.072–10.780	0.038
Cut vessel 1mm or larger in biopsy specimen (vs none)*	52.9% (9/17)	20.2% (18/89)	4.437	1.501–13.116	0.007
Cut bronchial structure in biopsy specimen (vs none)*	47.1% (8/17)	22.5% (20/89)	3.067	1.047–8.982	0.041

Note–Except where indicated, data are no. (%) of patients undergoing PTNB. OR = odd ratio, CI = confidence interval

* We exclude 5 cases in which no sample exists on the analysis of the cutting injury assessed base on the histopathologic evaluation

**Fig 2:**
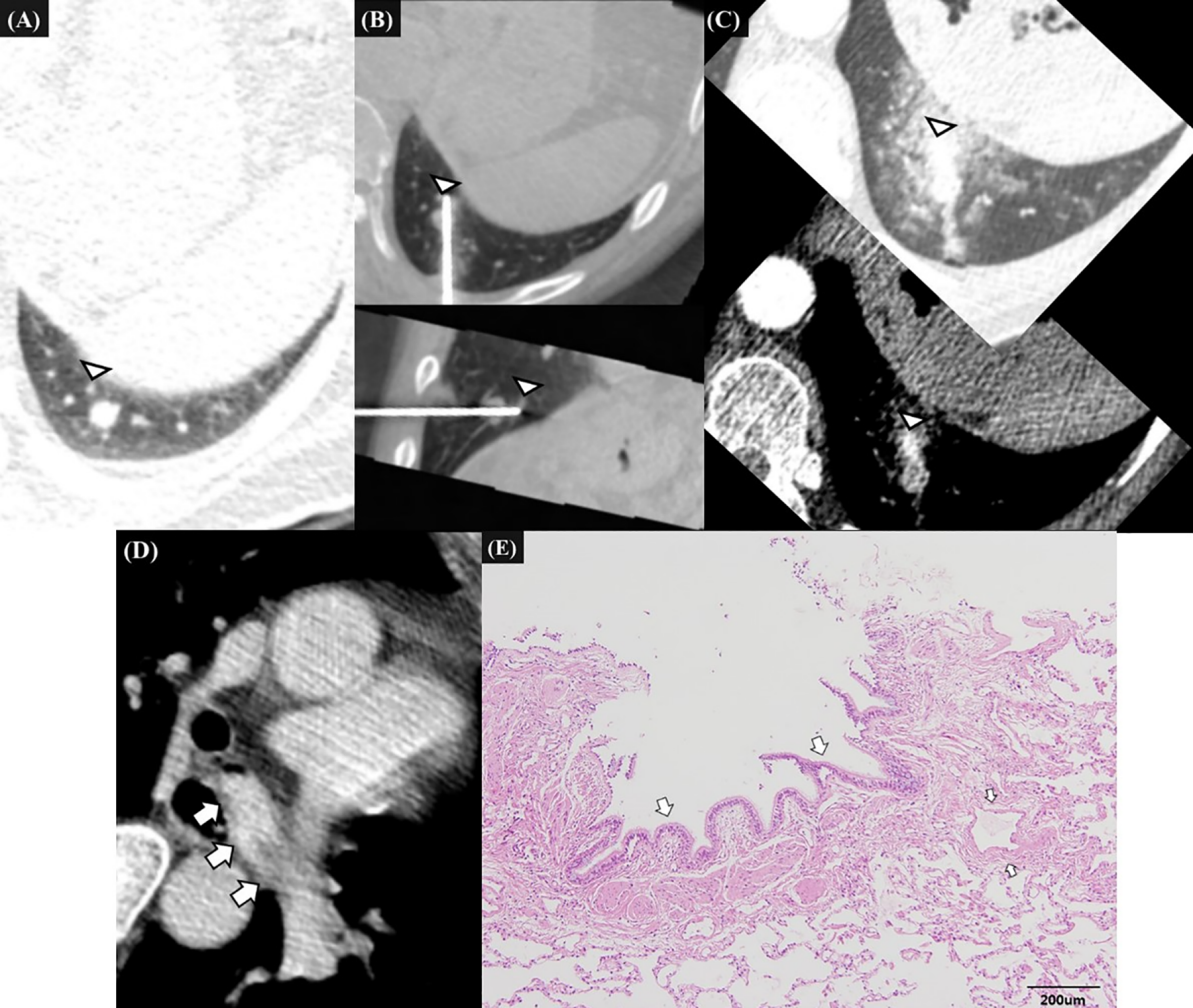
59-year-old male patient with hepatocellular carcinoma, representative case of clinically significant hemoptysis due to penetrating injury of peripheral pulmonary artery and cutting injury of bronchial structure after firing of biopsy gun. **A.** Pre-procedural CT image shows a 10mm-sized subpleural nodule in the left lower lobe which was suspected of lung metastasis from hepatocellular carcinoma. Small peripheral pulmonary vessel (white arrowhead) is located just behind the nodule. **B.** Intra-procedural transverse and sagittal CT images before biopsy show that the introducer needle penetrates the nodule. The vessel abuts the tip of introducer needle (white arrowheads) but does not lie along the expected track of biopsy gun. After pulling out the needle 1cm backwards, biopsy was performed once. After the firing, hemoptysis began abruptly. **C., D.** Transverse enhanced CT images 20 minutes after the onset of hemoptysis confirm an extravasation of contrast media from the peripheral small pulmonary artery. The extravasated contrast media filled the left main bronchus (white arrows). The patient was managed conservatively. Hemoptysis persists for 2.5 hours and then spontaneously decreased. **E.** The histopathologic examination of biopsy specimen show bronchial epithelium (white arrows) but not pulmonary vessel 1mm or larger.

**Fig 3:**
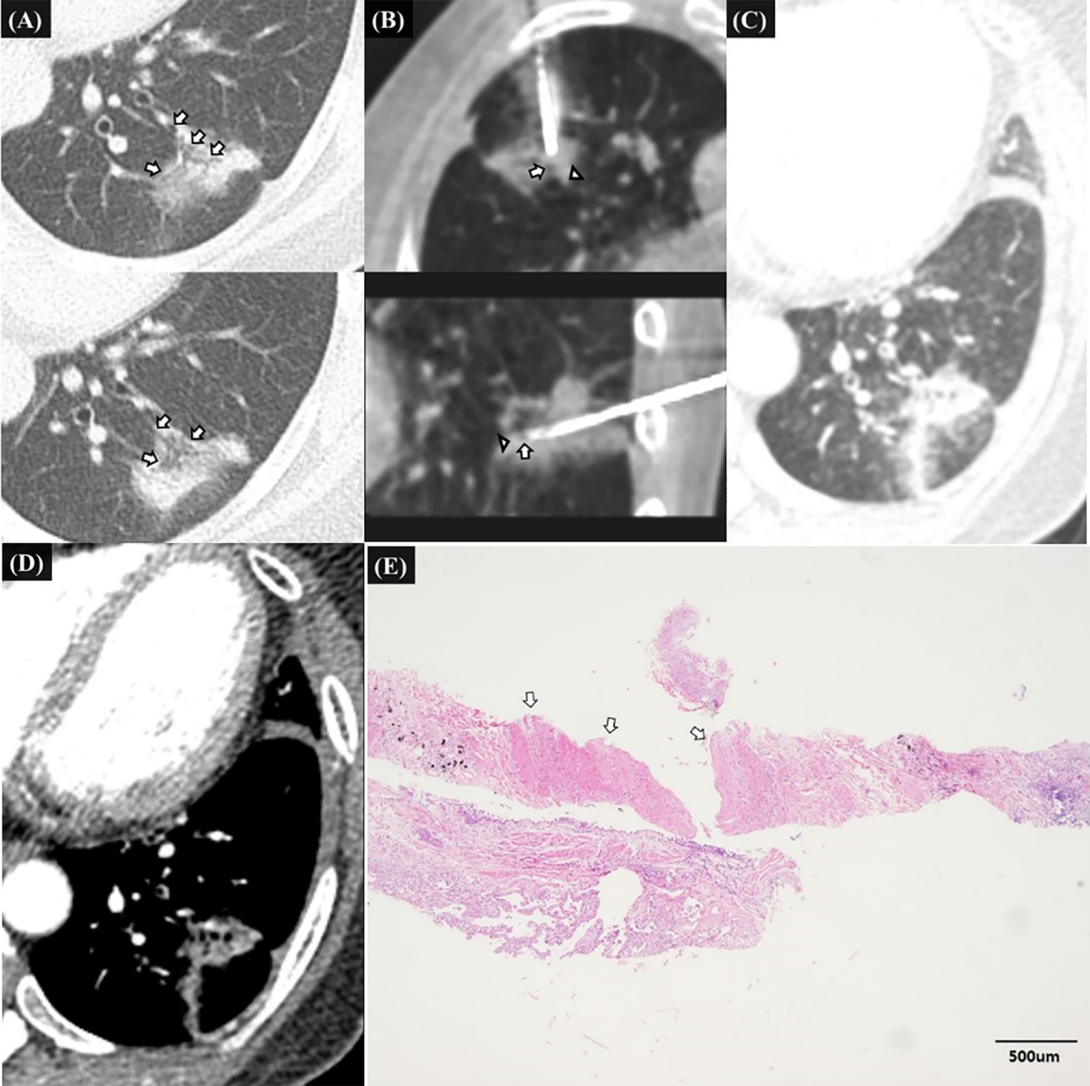
52-year-old female patients without specific medical history, representative case of clinically significant hemoptysis due to penetrating injury of pulmonary artery and bronchiole after firing of biopsy gun. **A.** Pre-procedural CT image shows a 32mm-sized sub solid mass in the left lower lobe with open bronchus sign (white arrow) which was suspected of primary lung cancer. **B.** Intra-procedural transverse and sagittal CT images before biopsy show that the introducer needle tip located within the mass. The bronchiole (white arrowhead) located just behind the introducer needle tip along the expected needle path of biopsy gun in transverse and sagittal CT images. The vessel (white arrow) which was seen in the medial margin of the mass lie along the expected track of biopsy gun in sagittal CT images. Biopsy was performed once and after the firing, hemoptysis began abruptly. **C., D.** Transverse enhanced CT image 20 minutes after the onset of hemoptysis shows the parenchymal hemorrhage along the introducer track. There was no evidence of extravasated contrast media around the mass. The patient was managed conservatively. **E.** The histopathologic examination of biopsy specimen shows pulmonary vessel larger than 1mm (white arrows) and small piece of bronchial epithelium. The pathologic diagnosis was consistent with primary lung adenocarcinoma.

**Fig 4:**
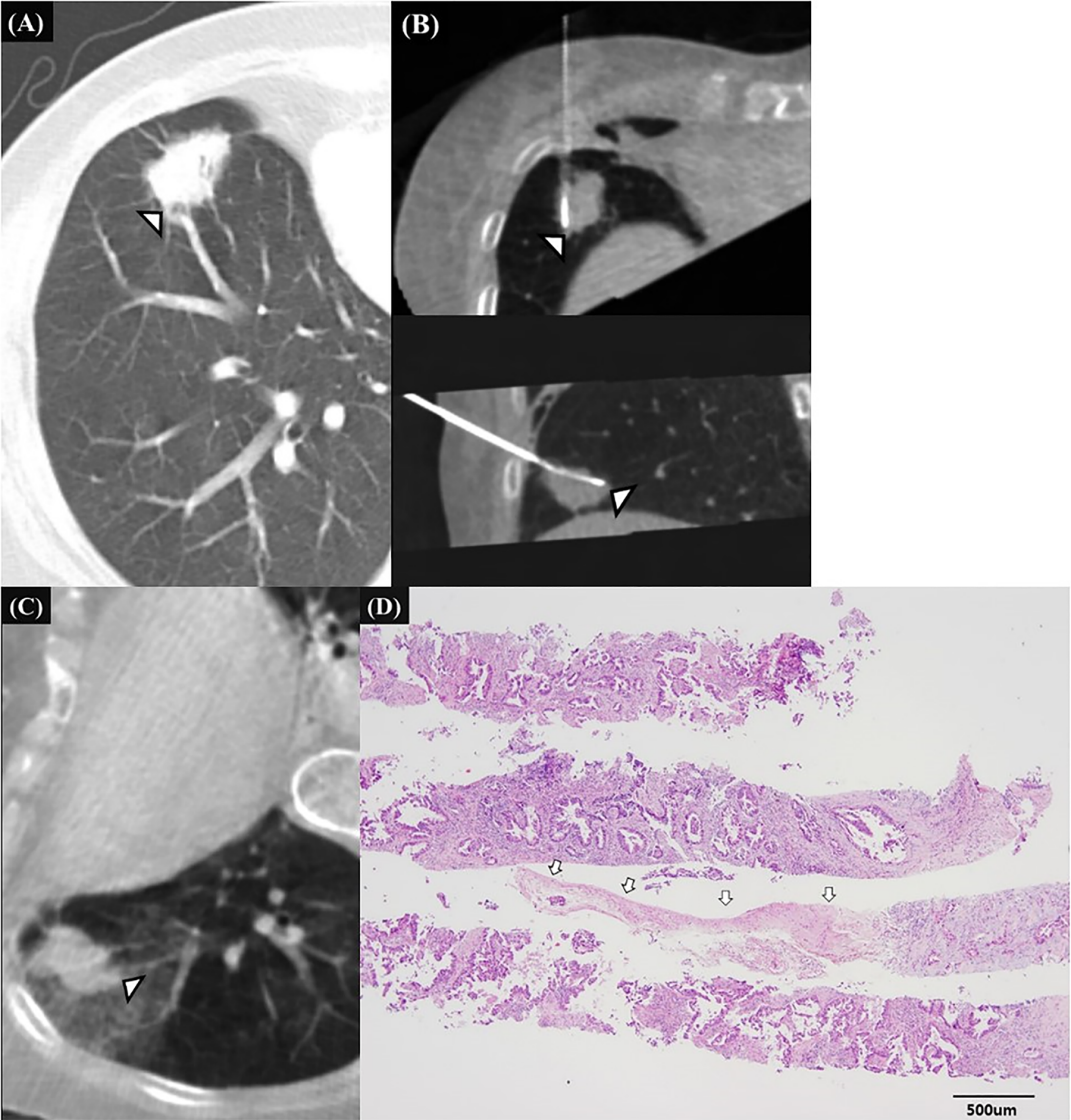
70-year-old female patients with history of pulmonary tuberculosis and cervical cancer, representative case of clinically significant hemoptysis due to penetrating injury of pulmonary artery after the firing of biopsy gun. **A.** Pre-procedural CT image shows a 22mm-sized spiculated nodule right lower lobe with air-bronchogram sign which was suspected of primary lung cancer. Small peripheral pulmonary vessel (white arrowhead) is located posterolateral margin of the mass. **B.** Intra-procedural transverse and sagittal CT images before biopsy show that the introducer needle tip located within the mass. The vessel (white arrowhead) located just behind the introducer needle tip along the expected needle path of biopsy gun. Biopsy was performed once and after the firing, hemoptysis began abruptly. **C.** Post-procedural transverse CT image with right decubitus position shows the parenchymal hemorrhage around the vessel (white arrow) expected to be damaged. **D.** The histopathologic examination of biopsy specimen show pulmonary vessel larger than 2mm (white arrows) but no bronchial structure was seen.

Multivariate analysis revealed that the potential penetration of a pulmonary vessel 1 mm or larger at firing (odd ratio [OR], 3.874; 95%CI, 1.072–13.997; *P* = .039), and a cutting injury of a pulmonary vessel 1 mm or larger (OR, 6.920; 95%CI, 1.728–27.711; *P* = .006) or a bronchial structure (OR, 4.604; 95%CI, 1.194–17.755; *P* = .027) were significantly associated with clinically significant hemoptysis (Table 2, Figs 2–4).

**Table 2:** Results of multivariate analysis to determine influencing factors for severity of hemoptysis.

Variables	OR	95% CI	P value
Subsolid nodule	1.070	0.186–6.153	0.940
Open bronchus sign	3.203	0.635–16.144	0.158
Potential penetration of vessel 1mm or larger when fired	3.874	1.072–13.997	0.039
Potential penetration of bronchus 1mm or larger when fired	1.432	0.286–7.154	0.662
Cut vessel 1mm or larger in biopsy specimen	6.920	1.728–27.711	0.006
Cut bronchial structure in biopsy specimen	4.604	1.194–17.755	0.027

Note—OR = odd ratio, CI = confidence interval

## Discussion

Our study found that clinically significant hemoptysis occurred in 15.3% cases after PTNB (17 of 111; 95%CI, 9.7%-23.2%) and was associated with bronchovascular injuries that occurred when a biopsy gun was fired, including the potential penetration of a pulmonary vessel 1 mm or larger at firing and cutting injuries of bronchovascular structures in biopsy specimens.

We evaluated the penetration of bronchovascular structures 1mm or larger on pre- and intra-procedural CBCT images that could be appropriately assessed given the spatial resolution of modern CT scans. In patients with hemoptysis, penetration was prevalent during the PTNB procedure: prior to firing, the penetration occurred along the path of needle insertion in 61% of patients, and potential penetration at firing was found in 47% of the patients. Nevertheless, the former type of penetration was not associated with clinically significant hemoptysis, whereas the latter type of penetration was significantly associated with clinically significant hemoptysis. This might originate from the difference in the physical impact of penetrating injuries. In general, a practitioner gently inserted the introducer needle into the lung parenchyma, but at the firing of the biopsy gun, the cutting needle advanced abruptly and strongly through a limited distance to obtain the biopsy specimen. The latter type of penetration can cause more extensive and devastating injuries (Fig 2).

A cutting injury of the bronchovascular structures was detected in 47.2% of our patients and was found to be the strongest associated factor for clinically significant hemoptysis. It can be reasonably expected that the extravasation of blood from cut pulmonary vessels directly results in a considerable amount of bleeding, and any hemorrhage from the cut bronchus directly flows out into the proximal airway, causing hemoptysis. The acquisition of specimens using a cutting needle is inevitably accompanied by varying degrees of cutting injuries of bronchovascular structures from the submillimeter to subcentimeter scale in diameter. Nevertheless, cutting injuries of bronchovascular structures of a certain diameter 1 mm or larger for pulmonary vessels seems to be responsible for clinically significant hemoptysis (Table 2).

Interestingly, cutting injuries of bronchovascular structures were present in approximately one-fifth of patients who did not have clinically significant hemoptysis. This discrepancy might originate from our binary definition of clinically significant hemoptysis. Of the patients who were categorized as having mild hemoptysis, those with cutting injuries of bronchovascular structures may have had a larger amount of hemoptysis than the other patients. We expect that this discrepancy would be potentially resolved if the amount of hemoptysis was precisely measured.

The risk of clinically significant hemoptysis can be reduced by avoiding bronchovascular injuries during the PTNB procedure. For this purpose, it is important to obtain thin-section pre-procedural CT images in a multi-planar reconstruction and to scrupulously review the intra-lesional and surrounding bronchovascular structures. The safe region of the biopsy target and the safe needle path for PTNB can be planned prior to the procedure. The needle can be inserted as planned with advanced CT-based guidance modalities. Nevertheless, a risk-benefit analysis of preoperative PTNB should also be conducted. If the cancer probability is sufficiently high, surgical resection can be performed without any exposure to the potential complications of PTNB [19].

In previous studies, factors such as deeper-located lesions, small lesions, older age, female gender, emphysema, dual-antiplatelet therapy (1-week discontinuation before PTNB in this study), and a large diameter of the cutting needle were reported to be risk factors for hemoptysis after PTNB [5–8, 20–23]. These risk factors did not show any significant associations in our study, implying that these factors may not affect the severity of hemoptysis, although these factors are associated with the occurrence of hemoptysis. Future studies are warranted to confirm the relationship of a priori known risk factors for the occurrence of hemoptysis with the severity of hemoptysis.

This study had several limitations. First, we did not analyze the presence of bronchovascular injuries in patients without hemoptysis and a lack of control group without any hemoptysis is the major limitation of our study. Because of the substantial spending of time and resources, our pathologists could not practically review the biopsy specimens in patients without hemoptysis. The causative association of bronchovascular injury with clinically significant hemoptysis after PTNB would be more convincing if the bronchovascular injury is scarce in a control group. We believe that our observation needs to be validated with further study which includes patients with or without hemoptysis in a prospective manner. Second, our study had a retrospective design with a limited number of patients. Third, since PTNB was performed by several radiologists, differences in experience and techniques across practitioners’ may be a confounder. Fourth, the potential penetration of bronchovascular structures at firing may not have always resulted in actual penetration because the practitioner may have attempted to tilt or bend the biopsy gun at firing (Fig 2). Nevertheless, since it is impractical to acquire CBCT images immediately after firing to assess the location of the fired gun at every launch of the biopsy gun, we believe that our tentative analysis of potential penetration was an adequate method of identifying penetrating injuries upon firing. Fifth, although we tried to define clinically significant hemoptysis precisely, a small proportion of patients who were classified as having mild hemoptysis might have actually had clinically significant hemoptysis. The impact of bronchovascular injuries on hemoptysis could be more clearly identified if patients who underwent PTNB are prospectively analyzed with accurate measurements of the amount of hemoptysis.

In conclusion, the potential penetration of a pulmonary vessel 1mm or larger on intra-procedural CBCT and a cutting injury of bronchovascular structures detected on pathologic specimens were independently associated with clinically significant hemoptysis after PTNB. Clinically significant hemoptysis is potentially preventable by avoiding bronchovascular injuries through a meticulous evaluation of bronchovascular structures 1 mm or larger on pre-procedural CT images as part of planning a safe needle path for PTNB in conjunction with advanced CT-based guidance modalities.

## Supporting information

S1 Fig Classification of biopsy specimen according to the maximum diameter of the included vascular structure. (a) Grade 2 includes vascular structures with a maximum diameter less than 1 mm. (b) Grade 3 includes vascular structures with a maximum diameter over 1mm and less than 1.5mm. (c) Grade 4 includes vascular structures with a maximum diameter over 1.5mm and less than 2mm. (d) Grade 5 includes vascular structures with a maximum diameter over 2mm.
(DOCX)

S2 Fig Classification of biopsy specimen according to presence or absence of bronchial structure. (a) Biopsy specimen containing bronchial epithelium. (b) Biopsy specimen containing cartilage (annotated as C).
(DOCX)

S1 Dataset Supporting information for acceptable data. (XLSX)
