# Supplementary material for: Bronchovascular injury associated with clinically significant hemoptysis after CT-guided core biopsy of the lung: Radiologic and histopathologic analysis
Source: PLoS One. 2018 Sep 21;13(9):e0204064. doi: 10.1371/journal.pone.0204064 (PMC6150475; doi:10.1371/journal.pone.0204064)

**Supporting information**

**S1 Fig. Classification of biopsy specimen according to the maximum diameter of the included vascular structure.**

(a) Grade 2 includes vascular structures with a maximum diameter less than 1 mm. (b) Grade 3 includes vascular structures with a maximum diameter over 1mm and less than 1.5mm. (c) Grade 4 includes vascular structures with a maximum diameter over 1.5mm and less than 2mm. (d) Grade 5 includes vascular structures with a maximum diameter over 2mm.


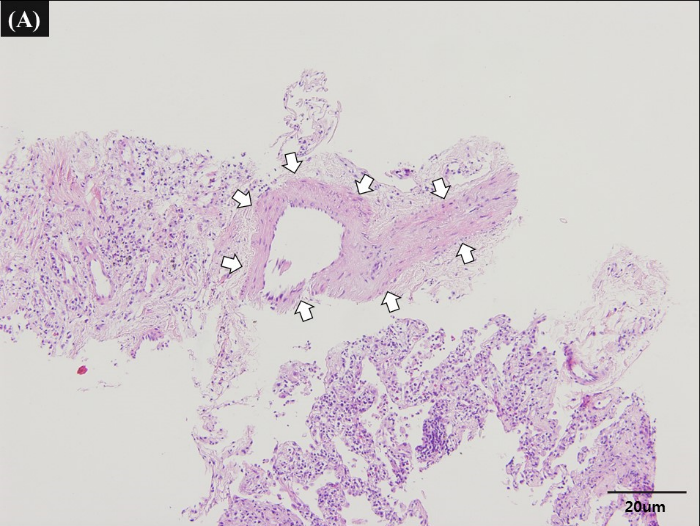

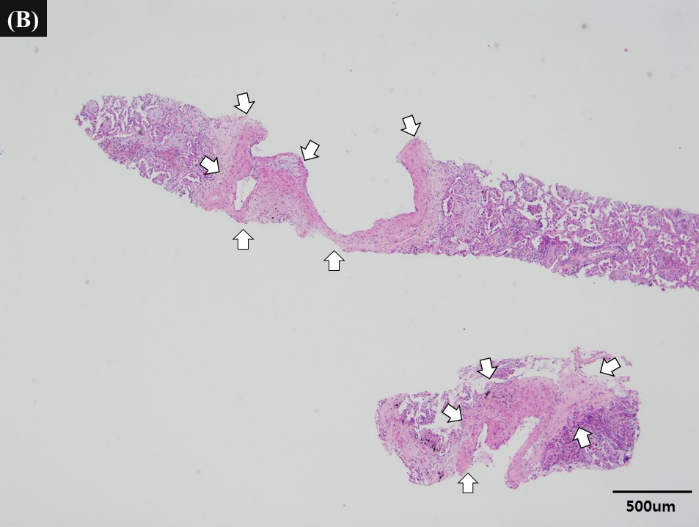

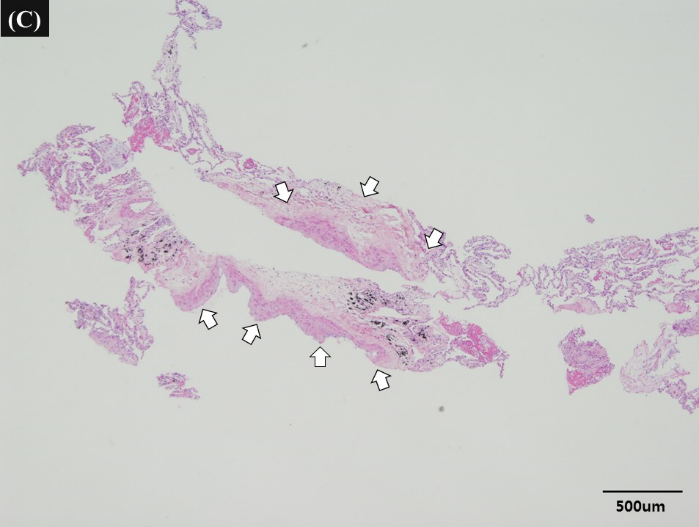

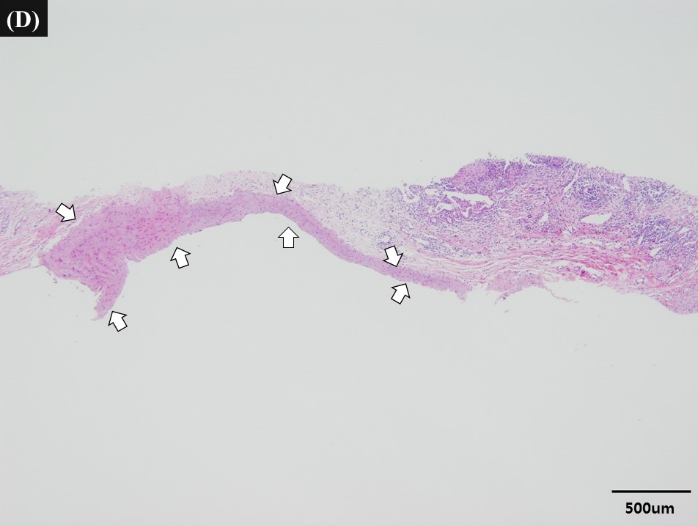

Supplement: S1 Fig — (a) Grade 2 includes vascular structures with a maximum diameter less than 1 mm. (b) Grade 3 includes vascular structures with a maximum diameter over 1mm and less than 1.5mm. (c) Grade 4 includes vascular structures with a maximum diameter over 1.5mm and less than 2mm. (d) Grade 5 includes vascular structures with a maximum diameter over 2mm. (DOCX) [file pone.0204064.s001.docx]
