# Supplementary material for: Bronchovascular injury associated with clinically significant hemoptysis after CT-guided core biopsy of the lung: Radiologic and histopathologic analysis
Source: PLoS One. 2018 Sep 21;13(9):e0204064. doi: 10.1371/journal.pone.0204064 (PMC6150475; doi:10.1371/journal.pone.0204064)

**Supporting information**

**S2 Fig. Classification of biopsy specimen according to presence or absence of bronchial structure.**

(a) Biopsy specimen containing bronchial epithelium. (b) Biopsy specimen containing cartilage (annotated as C).


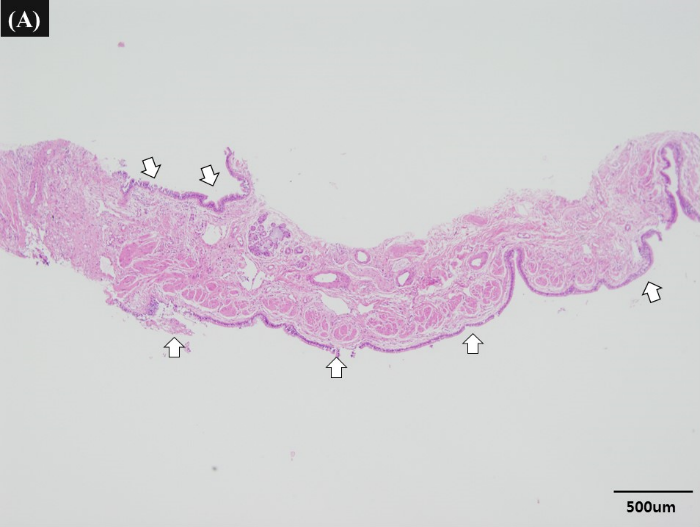

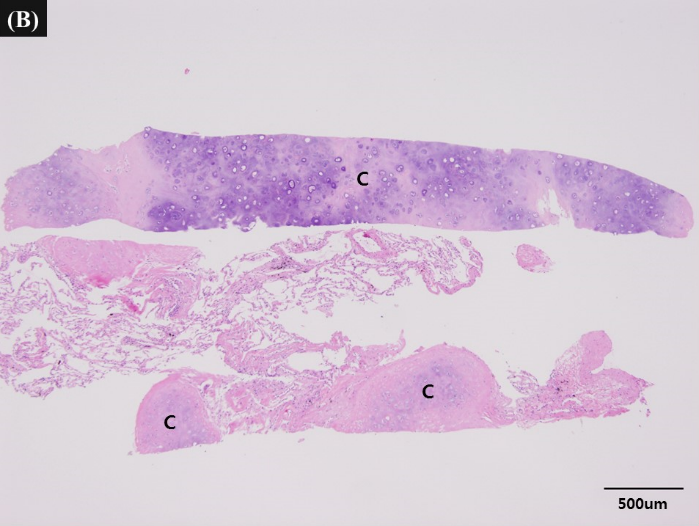

Supplement: S2 Fig — (a) Biopsy specimen containing bronchial epithelium. (b) Biopsy specimen containing cartilage (annotated as C). (DOCX) [file pone.0204064.s002.docx]
